# 

**Published:** 1916-12

**Authors:** 


### Publishers' Announcement

A little submarine can sink a five million dollar battle ship; a penny by wise manipulation can grow to be a fortune, and a smal advertisement can bring returns far in excess of its cost. The cause of these phenomena is ad—vantage. The submarine can not be seen until too late. The penny has wise and careful care. The advertisement is placed in a medium which is a recognized help to its readers.

The DENTAL REGISTER soon enters upon its seventy-first year of continuous publication. If age stands for any enduring qualities, the DENTAL REGISTER may surely lay claim to the recognition of the dental world as a faithful disciple of truthful advertising and diligent worker in dental literature and comment. Dear old Jonathan Taft's work still goes on in the kind and thoughtful hands of Nelville S. Hoff and dear old Sam'l A. Crocker's work goes on in the efficient management by the corporation which he founded and which bears his name. The Sam'l A. Crocker Co.

